# A cross-sectional investigation of trend in career specialty preference among clinical year medical undergraduates, including factors influencing preferences and discouragement

**DOI:** 10.3389/fmed.2025.1665043

**Published:** 2025-10-17

**Authors:** Muhammad Hamza Dawood, Filza Mir, Hajrah Hilal Ahmed, Mehmooda Wasim, Muniba Athar Khan, Ali Hasan, Umair Ul Islam, Raheel Ahmed

**Affiliations:** ^1^Department of Surgery, United Medical and Dental College, Karachi, Pakistan; ^2^Department of Medicine, United Medical and Dental College, Karachi, Pakistan; ^3^Department of Medicine, Karachi Medical and Dental College, Karachi, Pakistan; ^4^Imperial College, London, United Kingdom; ^5^National Heart and Lung Institute, Imperial College, London, United Kingdom; ^6^Department of Cardiology, Newcastle University, Newcastle upon Tyne, United Kingdom; ^7^Department of Cardiology, University of Sunderland, Sunderland, United Kingdom; ^8^Freeman Hospital, Newcastle upon Tyne, United Kingdom

**Keywords:** career selection, preference, discouragement, medical students, medical specialty, surgery, medicine, Pakistan

## Abstract

**Introduction:**

Selecting a medical specialty is a crucial decision influenced by personal, professional, and societal factors. However, data on these determinants among clinical-year medical students in Pakistan remain scarce. This study seeks to identify the specialty preferences of clinical-year medical undergraduates in Pakistan and to elucidate the factors influencing their preference and discouragement decisions.

**Methods:**

This investigation employed a cross-sectional survey methodology involving clinical-year medical students from September 16th, 2024, to November 10th, 2024 among both private and public medical colleges in Karachi. A total of 436 participants were randomly selected using a simple random sampling technique. Chi-square/Fisher’s Exact tests, were performed to analyze trends in career preferences, assess the association between specialty preferences and demographic variables, and identify factors influencing career preference and discouragement, both generally and within specific specialties and demographic categories.

**Results:**

Among the 430/436 respondents, 28.6% were male and 71.4% were female. 56.9% of males preferred surgery, while 52.7% of females favored medicine as their career choice. Students from private institutions exhibited a higher preference for surgery (51.3%) compared to government institution students, who favored medicine (53.1%). A notable preference for surgery was observed among 3rd-year students (37.2%), with a significant shift towards medicine by 5th-year students (42.8%) (P-value=0.002). The principal factors affecting career preference were own interest/passion (85%), vast-career opportunities (31.1%), and clinical rotations (29.7%). Major discouragement factors included lack of mentorship (76.3%), gender inequity (46.7%), and family obligations (31.6%). A comparable trend was observed across specialty, year of education, sex, and institutional sector for both career preference (P-values: 0.014, 0.000, 0.274, 0.011) and discouragement factors (Pvalues: 0.000, 0.828, 0.150, 0.000).

**Discussion:**

The findings underscore key challenges in medical students’ specialty choices, including significant mentorship gaps, pervasive gender inequity, and family obligations. Addressing these issues is essential for improving career decision-making and ensuring a more balanced distribution of specialties within Pakistan’s medical workforce. Targeted interventions are needed to mitigate these barriers and support students in making informed career decisions.

## 1 Introduction

Choosing a medical specialty is a complex and pivotal decision for medical undergraduates, significantly impacting their future career trajectory and the composition of the healthcare workforce (1–3). In recent years, there has been increasing interest in understanding the career specialty preferences of medical students, with studies highlighting the dynamic interplay of choices between various specialties influenced by various factors within the evolving medical landscape (4–10). These factors are vital in shaping the future medical workforce, and their impact has been increasingly acknowledged over the past decade (4, 11, 12). Prior literature suggests that students often vary in their inclination toward medicine or surgery, with multiple determinants shaping their decisions (5–10). These determinants include personal and professional development, work-life balance, interest in the specialty, flexibility, income, job security, demographic characteristics, influence of role models, family considerations, career objectives, future prospects, and cultural and societal factors (2, 3, 6, 7, 9–16). However, findings across studies remain inconsistent, particularly for clinical-year students who are nearing definitive career decisions.

Despite the abundance of global literature, there remains a notable gap in the context of Pakistan (17, 18). Existing local evidence indicates that while surgery is often preferred during undergraduate training (17), post-graduation career choices often diverge (19), with gender inequity identified as a significant factor influencing this shift (20). With the exponential growth of the population and the corresponding demand for healthcare services in low-middle-income country, an imbalanced distribution of doctors across specialties could exacerbate workforce shortages. Understanding the determinants of specialty selection among clinical-year students is therefore critical, as these decisions will directly shape the future medical workforce and influence healthcare delivery.

Therefore, our present study endeavors to address this gap by focusing on clinical-year medical undergraduates in Pakistan, through following objectives: To ascertain the predominant specialty preferences among clinical year medical undergraduates. To evaluate the trend of predominant specialty preferences according to demographic characteristics of clinical year medical undergraduates. To explore the factors influencing career specialty preferences and discouragement among clinical year medical undergraduates. To examine the influencing factors behind career specialty preferences and discouragement, based on specialty and demographic characteristics of clinical year medical undergraduates.

## 2 Materials and methods

### 2.1 Study setting, design and period

This cross-sectional study was conducted from September 16th, 2024, till November 10th, 2024, involving clinical-year medical students (third, fourth, and fifth years) from both private and government medical colleges in Karachi, Pakistan, after getting Ethical approval from IRB committee of United Medical and Dental College (UMDC).

### 2.2 Sample size and sampling techniques

We calculated a sample size of 357 utilizing the Openepi finite sample size formula (*n* = deff Npq/[d^2/1.96]^2(N − 1) + pq), applying a 95% confidence level and a 5% margin of error. This estimation was based on a population size of approximately 5,000 clinical-year students from government and private medical colleges in Karachi, which confer a Bachelor of Medicine and Bachelor of Surgery (MBBS) degree accredited by the Pakistan Medical and Dental Council (PMDC). To enhance statistical robustness, the final sample size was increased to 436, ensuring a minimum of 26 participants from each college. We implemented a simple random probability sampling method, selecting students randomly from a compiled list using the Giga calculator random name selector tool (21), which helped mitigate selection and participation bias. The student list was compiled with assistance from each college’s students, and the giga random selector tool was chosen for its ease of use and to maintain the integrity of the selection process, avoiding potential repetition issues associated with other tools like Excel.

### 2.3 Inclusion and exclusion criteria

The inclusion criteria for the study encompassed undergraduate medical students in their clinical years of the MBBS program, aged between 18 and 30 years, who were enrolled in government and private medical colleges in Karachi. Exclusion criteria were applied to first- and second-year MBBS students, as they typically lack sufficient clinical exposure and familiarity with the study’s subject matter. Furthermore, individuals with documented psychological disorders or those who did not provide written consent were excluded. Furthermore, individuals with documented psychological disorders or those who did not provide written consent were excluded. This methodology was designed to preserve data integrity, adhere to ethical standards, and safeguard participant well-being, as the inclusion of individuals with psychological conditions could potentially aggravate their conditions during discussions of related issues.

### 2.4 Study tool, instrument validity and data collection procedure

A self-administered, semi-structured questionnaire, accompanied by a consent form, was employed in this study. The questionnaire, developed by the authors, was informed by insights from previous research (2–17) (Supplementary Appendix A). To establish its validity, a panel of five Ethics Board members among them three having expertise in medical education reviewed the instrument, focusing on aspects such as validity, homogeneity, double-barreled questions, and potential writing or grammatical errors. Prior to the main study, a pilot study was conducted with 30 students, 10 from each academic year, to evaluate the questionnaire’s timing, validity, and clarity. The questionnaire was divided into two sections: Section I included five questions pertaining to demographic characteristics, while Section II comprised three questions related to career specialty preference and the factors influencing these preferences and discouragement. The survey process commenced with obtaining written informed consent, followed by a verbal briefing on the authors, study objectives, participant involvement, and the implications of withdrawal or non-participation. Data collection was conducted face-to-face during students’ college hours to optimize response rates and ensure participants’ comprehension of the questionnaire.

### 2.5 Statistical analysis procedure

Responses were documented in an Excel spreadsheet and subsequently imported into IBM SPSS version 23 for analysis. Frequencies and percentages are reported for categorical variables. The Chi-square test or Fisher’s Exact test was employed to assess associations or differences between variables. Statistical significance was determined at a *p*-value threshold of <0.05. Cramer’s V was applied for effect sizes.

### 2.6 Ethical consideration

Ethical approval for the study was obtained from the IRB committee of UMDC (UMDC/ETHICS/2024/16/09/364). The research was conducted in accordance with the latest version of the Declaration of Helsinki for studies involving human participants. Additionally, access to the compiled student list during selection and data collection was restricted solely to the authors. To ensure confidentiality and anonymity, all personal identifiers including names, email addresses, and institutional affiliations were removed. Only non-identifiable data on general demographics, career specialties, and related factors were utilized for analysis.

### 2.7 Generative artificial intelligence

ChatGPT, Grammarly, and Quillbot, were employed to assist with paraphrasing, correction of grammatical errors, and refinement of the scientific tone.

## 3 Results

### 3.1 Demographic data of participants

Among the 436 participants approached, 430 completed the questionnaire, yielding a response rate of 98%. The majority of participants were female, with most affiliated with the private sector. Both male and female participants were aged between 18 and 26 years, with an equal distribution across clinical years observed among the cohort (Table 1).

**Table 1:** Demographic characteristic of participants (*N* = 430).

Variable	Respondents *n* (%)
Sex	
Male	123 (28.6%)
Female	307 (71.4%)
Current education status	
3rd year	129 (30.0%)
4th year	146 (34.0%)
5th year	155 (36.0%)
Sector	
Government sector	128 (29.8%)
Private sector	302 (70.2%)

### 3.2 Trend in career preferences

Overall, the participating members exhibited an equal distribution of career preference between surgery (50%) and medicine (50%) (*p*-value = 1). A notable preference for surgery was observed among 3rd-year students, with a balanced distribution in 4th-year students and a marked shift of trend towards medicine by 5th-year students relative to their third-year counterparts. Sex-based preferences indicated a greater inclination for surgery among male students and a stronger preference for medicine among female students. Furthermore, students from private institutions demonstrated a higher preference for surgery compared to their peers from government institutions (Table 2).

**Table 2:** Trends in career preferences of respondents among clinical year undergraduates based on demographic characteristics (*N* = 430).

Variable	Respondents		*p*-value (Chi-square)	Cramer’s V
	Surgery *n* (%)	Medicine *n* (%)		
Current education status				
3rd year	80 (37.20%)	49 (22.80%)	0.002 (12.903)	0.2
4rth year	72 (33.50%)	74 (34.40%)		
5th year	63 (29.30%)	92 (42.80%)		
Sex				
Male	70 (32.6%)	53 (24.7%)	0.070 (3.291)	0.1
Female	145 (67.4%)	162 (75.3%)		
Institutional sector				
Government	60 (27.9%)	68 (31.6%)	0.399 (0.712)	0.0
Private	155 (72.1%)	147 (68.4%)		

### 3.3 Factors influencing career preference and discouragement

**Figure 1:**
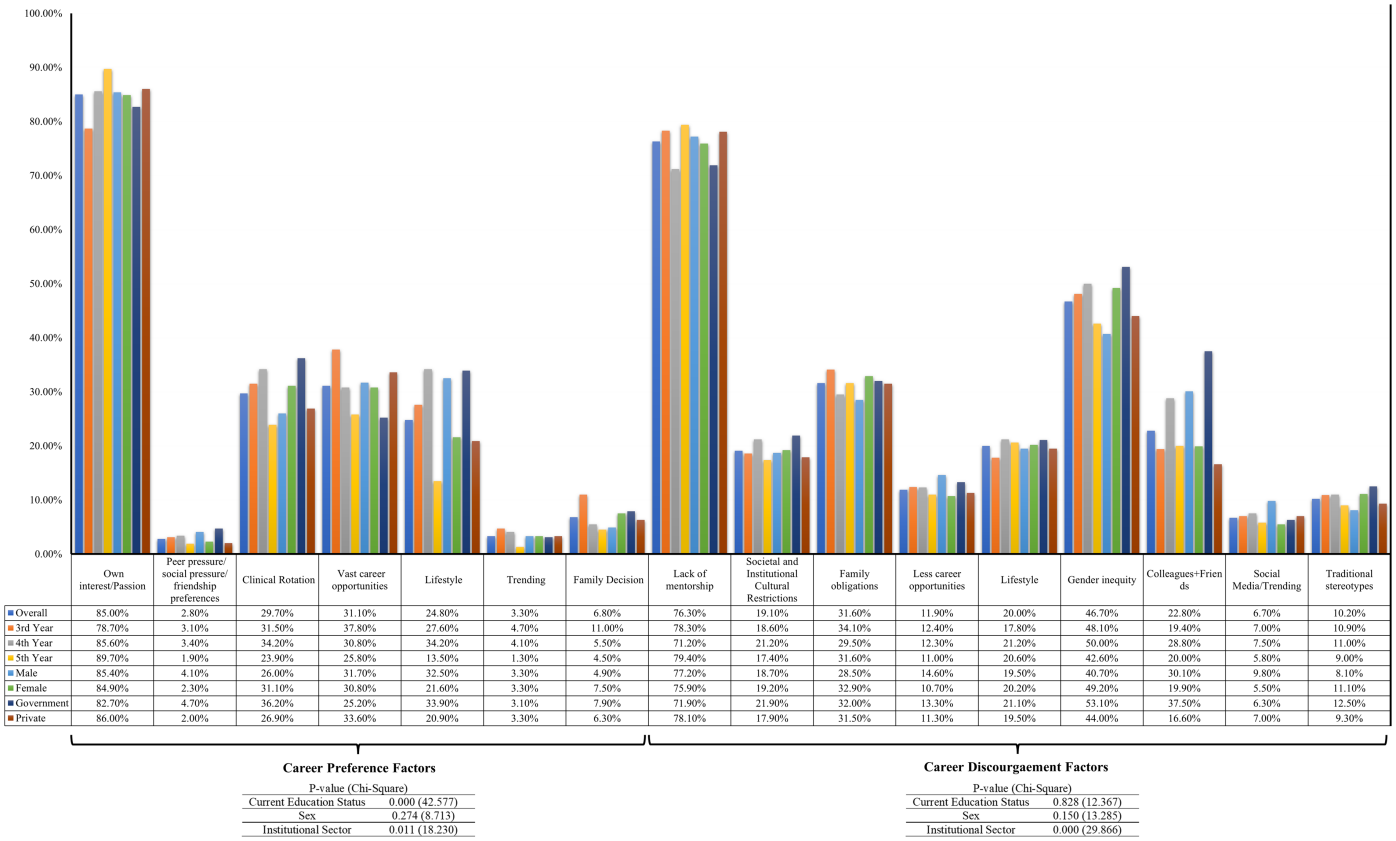
Factors influencing career preference and discouragement among clinical year undergraduates (*N* = 430). This figure presents the distribution of motivating and discouraging factors influencing specialty choices among clinical-year undergraduate medical students in Karachi, Pakistan. Motivating factors included personal interest, mentorship, peer influence, perceived career opportunities, and exposure during clinical rotations. Discouraging factors encompassed lack of mentorship, gender inequity, family obligations, and lifestyle considerations. Data were obtained from a structured questionnaire administered to third-, fourth-, and fifth-year MBBS students from both public and private institutions. Percentages indicate the proportion of students reporting each factor relative to the total study population.

During the clinical year, several pivotal factors were identified as influencing both career preferences and discouragement among students. Career preference was predominantly driven by own interest/Passion, vast career opportunities, and clinical rotations. Conversely, factors significantly contributing to career discouragement included lack of mentorship, gender inequity, and family obligations. A similar trend was observed across education year, sex, and institutional sector, with the exception that for students from government institutions, colleagues and friends emerged as the second most significant discouragement factor (Figure 1). In terms of specialty, the three primary factors influencing a career in surgery were own interest/passion, vast career opportunities, and clinical rotations, whereas, for medicine, it was own interest/passion, lifestyle, and clinical rotations. The three most significant discouragement factors for both surgery and medicine included a lack of mentorship, gender inequity, and family obligations (Table 3). A similar trend was observed across education year, sex, and institutional sector for career preference (Table 4) and discouragement (Table 5), except that for students from government institutions, colleagues and friends emerged as the second most significant discouragement factor for pursuing a career in surgery.

**Table 3:** Factors influencing career preference and discouragement among clinical year undergraduates, categorized by specialty (*N* = 430).

Variable	Respondents		*p*-value (Chi-square)	Cramer’s V
	Surgery *n* (%)	Medicine *n* (%)		
Factors influencing career preference				
Own interest/Passion	188 (88.3%)	176 (81.9%)	0.014 (17.627)	0.2
Mentorship	0 (0.0%)	0 (0.0%)		
Peer/social pressure and preferences	3 (1.4%)	9 (4.2%)		
Clinical rotation	64 (30.0%)	63 (29.3%)		
Vast career opportunities	71 (33.3%)	62 (28.8%)		
Lifestyle	41 (19.2%)	65 (30.2%)		
Trending	6 (2.8%)	8 (3.7%)		
Family decision	10 (4.7%)	19 (8.8%)		
Factors influencing career discouragement				
Lack of mentorship	178 (82.8%)	150 (69.8%)	0.000 (38.303)	0.3
Societal/Institutional/Cultural norms	28 (13.0%)	54 (25.1%)		
Family obligations	68 (31.6%)	68 (31.6%)		
Less career opportunities	22 (10.2%)	29 (13.5%)		
Lifestyle	29 (13.5%)	57 (26.5%)		
Gender inequity	108 (50.2%)	93 (43.3%)		
Colleagues/Friends	50 (23.3%)	48 (22.3%)		
Social media trends	19 (8.8%)	10 (4.7%)		
Traditional stereotypes	20 (9.3%)	24 (11.2%)		

**Table 4:** Factors influencing career preference among clinical year undergraduates, dissected by specialty with demographic characteristics (*N* = 430).

Variable/Group			Factors influencing career preference									
			Own interest/Passion	Mentorship	Peer/Social pressure	Clinical rotation	Vast career opportunities	Lifestyle	Trending	Family decision	*p*-value (Chi-square)	Cramer’s V
Current education status	3rd year	Surgery	65 (83.3%)	0 (0.0%)	2 (2.6%)	26 (33.3%)	31 (39.7%)	15 (19.2%)	4 (5.1%)	5 (6.4%)	0.037 (14.901)	0.2
		Medicine	35 (71.4%)	0 (0.0%)	2 (4.1%)	14 (28.6%)	17 (34.7%)	20 (40.8%)	2 (4.1%)	9 (18.4%)		
	4th year	Surgery	64 (88.9%)	0 (0.0%)	1 (1.4%)	23 (31.9%)	17 (23.6%)	20 (27.8%)	1 (1.4%)	3 (4.2%)	0.083 (12.595)	0.2
		Medicine	61 (82.4%)	0 (0.0%)	4 (5.4%)	27 (36.5%)	28 (37.8%)	30 (40.5%)	5 (6.8%)	5 (6.8%)		
	5th year	Surgery	59 (93.7%)	0 (0.0%)	0 (0.0%)	15 (23.8%)	23 (36.5%)	6 (9.5%)	1 (1.6%)	2 (3.2%)	0.93 (12.238)	0.2
		Medicine	80 (87.0%)	0 (0.0%)	3 (3.3%)	22 (23.9%)	17 (18.5%)	15 (16.3%)	1 (1.1%)	5 (5.4%)		
Sex	Male	Surgery	62 (88.6%)	0 (0.0%)	2 (2.9%)	20 (28.6%)	23 (32.9%)	17 (24.3%)	1 (1.4%)	2 (2.9%)	0.149 (10.761)	0.2
		Medicine	43 (81.1%)	0 (0.0%)	3 (5.7%)	12 (22.6%)	16 (30.2%)	23 (43.4%)	3 (5.7%)	4 (7.5%)		
	Female	Surgery	126 (88.1%)	0 (0.0%)	1 (0.7%)	44 (30.8%)	48 (33.6%)	24 (16.8%)	5 (3.5%)	8 (5.6%)	0.121 (11.420)	0.2
		Medicine	133 (82.1%)	0 (0.0%)	6 (3.7%)	51 (31.5%)	46 (28.4%)	42 (25.9%)	5 (3.1%)	15 (9.3%)		
Institutional sector	Government	Surgery	52 (88.1%)	0 (0.0%)	3 (5.1%)	20 (33.9%)	16 (27.1%)	12 (20.3%)	4 (6.8%)	0 (0.0%)	0.001 (25.969)	0.3
		Medicine	53 (77.9%)	0 (0.0%)	3 (4.4%)	26 (38.2%)	16 (23.5%)	31 (45.6%)	0 (0.0%)	10 (14.7%)		
	Private	Surgery	136 (88.3%)	0 (0.0%)	0 (0.0%)	44 (28.6%)	55 (35.7%)	29 (18.8%)	2 (1.3%)	10 (6.5%)	0.56 (13.740)	0.2
		Medicine	123 (83.7%)	0 (0.0%)	6 (4.1%)	37 (25.2%)	46 (31.3%)	34 (23.1%)	8 (5.4%)	9 (6.1%)		

**Table 5:** Factors influencing career discouragement among clinical year undergraduates, dissected by specialty with demographic characteristics (*N* = 430).

Variable/Group			Factors influencing career preference										
			Lack of mentorship	Societal/Cultural norms	Family obligations	Less career opportunities	Lifestyle	Gender inequity	Colleagues/Friends	Social media trends	Traditional stereotypes	*p*-value (chi-square)	Cramer’s V
Current education status	3rd year	Surgery	67 (83.8%)	12 (15.0%)	24 (30.0%)	8 (10.0%)	9 (11.3%)	37 (46.3%)	15 (18.8%)	8 (10.0%)	10 (12.5%)	0.032 (18.307)	0.2
		Medicine	34 (69.4%)	12 (24.5%)	20 (40.8%)	8 (16.3%)	14 (28.6%)	25 (51.0%)	10 (20.4%)	1 (2.0%)	4 (8.2%)		
	4th year	Surgery	54 (75.0%)	10 (13.9%)	21 (29.2%)	6 (8.3%)	9 (12.5%)	37 (51.4%)	21 (29.2%)	6 (8.3%)	7 (9.7%)	0.102 (14.620)	0.2
		Medicine	50 (67.6%)	21 (28.4%)	22 (29.7%)	12 (16.2%)	22 (29.7%)	36 (48.6%)	21 (28.4%)	5 (6.8%)	9 (12.2%)		
	5th year	Surgery	57 (90.5%)	6 (9.5%)	23 (36.5%)	8 (12.7%)	11 (17.5%)	34 (54.0%)	14 (22.2%)	5 (7.9%)	3 (4.8%)	0.004 (23.966)	0.2
		Medicine	66 (71.7%)	21 (22.8%)	26 (28.3%)	9 (9.8%)	21 (22.8%)	32 (34.8%)	17 (18.5%)	4 (4.3%)	11 (12.0%)		
Sex	Male	Surgery	57 (81.4%)	12 (17.1%)	21 (30.0%)	8 (11.4%)	8 (11.4%)	29 (41.4%)	22 (31.4%)	6 (8.6%)	4 (5.7%)	0.220 (11.878)	0.2
		Medicine	38 (71.7%)	11 (20.8%)	14 (26.4%)	10 (18.9%)	16 (30.2%)	21 (39.6%)	15 (28.3%)	6 (11.3%)	6 (11.3%)		
	Female	Surgery	121 (83.4%)	16 (11.0%)	47 (32.4%)	14 (9.7%)	21 (14.5%)	79 (54.5%)	28 (19.3%)	13 (9.0%)	16 (11.0%)	0.001 (35.671)	0.3
		Medicine	112 (69.1%)	43 (26.5%)	54 (33.3%)	19 (11.7%)	41 (25.3%)	72 (44.4%)	33 (20.4%)	4 (2.5%)	18 (11.1%)		
Institutional sector	Government	Surgery	47 (78.3%)	10 (16.7%)	15 (25.0%)	5 (8.3%)	8 (13.3%)	38 (63.3%)	28 (46.7%)	6 (10.0%)	8 (13.3%)	0.003 (24.733)	0.2
		Medicine	45 (66.2%)	18 (26.5%)	26 (38.2%)	12 (17.6%)	19 (27.9%)	30 (44.1%)	20 (29.4%)	2 (2.9%)	8 (11.8%)		
	Private	Surgery	131 (84.5%)	18 (11.6%)	53 (34.2%)	17 (11.0%)	21 (13.5%)	70 (45.2%)	22 (14.2%)	13 (8.4%)	12 (7.7%)	0.001 (27.829)	0.3
		Medicine	105 (71.4%)	36 (24.5%)	42 (28.6%)	17 (11.6%)	38 (25.9%)	63 (42.9%)	28 (19.0%)	8 (5.4%)	16 (10.9%)		

## 4 Discussion

In this study, we explored the specialty preferences and the factors influencing career specialty selection and discouragement among clinical-year medical students in Karachi, Pakistan. In doing so, we extend the predominantly Pakistani literature by situating our findings within a broader South Asian context. Our findings revealed distinct trends in specialty preferences as students advanced through their educational years, with variations observed across sex and institutional sector. These results highlight the evolving nature of specialty selection, suggesting that as student’s progress in their education, their preferences are influenced by a combination of personal, academic, and environmental factors. Such insights are essential for understanding how medical undergraduates make career decisions and can inform strategies to guide and support them in choosing their future specialties.

Career preferences in our cohort were evenly distributed between surgery and medicine overall, but the trajectory of choices shifted across academic years. Third-year students exhibited a pronounced preference for surgery, which transitioned to a more balanced distribution in the fourth year and shifted notably toward medicine by the fifth year. This trend aligns with findings from Khan Q et al., from Pakistan as well as with similar regions, Bangladesh and India, where surgery initially appeals to most medical students, medicine becomes the most favored specialty choice among medical students as they progress through their academic years (13, 22, 23). The similarity in findings can be attributed to the shared socio-cultural and economic contexts and influences between Pakistan, Bangladesh and India, and this shift in trend likely reflects the impact of clinical exposure, which shapes students’ perceptions of specialty demands, work-life balance, and the long-term viability of career choices (24–27). Additionally, with comparison across similar low- and middle-income countries (LMICs), from Jordan, Nigeria and Nepal early-year and clinical year students also favor surgical specialties, but their preferences for medicine increase over time (27–30). The early preference for surgery among junior and initial clinical year students across LMICs may be explained by the initial excitement, perceived prestige, and technical appeal associated with operative disciplines, coupled with limited early clinical exposure to medical specialties (31, 32). Surgery is often regarded as a “glamorous” field requiring skill-intensive training, which aligns with the aspirations of students at the beginning of their clinical journey (33–35). However, as students advance through their training, greater exposure to diverse rotations, mentorship experiences, and firsthand insight into workload, career opportunities, payouts and lifestyle demands contribute to a more pragmatic reassessment of career goals. Internal medicine, with its broader patient interaction, continuity of care, and comparatively favorable work–life balance, gradually becomes more attractive, tempering the initial enthusiasm for surgical careers (36). This progression underscores how evolving clinical exposure and professional realities reshape students’ specialty choices, transforming initial aspirations into more balanced and pragmatic career decisions.

Furthermore, in contrast, medical students from high-income countries tend to report a higher preference for surgery over medicine, throughout their medical career (32, 37). The disparity between low- and middle-income countries (LMICs) and high-income countries in specialty preference may stem from differences in healthcare infrastructure, resources, societal and cultural factors, and the perceived prestige of specialties, with surgery often viewed as more prestigious in wealthier nations and medicine as more practical in lower-income settings (33–36), a trend further supported by Puertas et al.’s study (38), which shows that LMIC medical students tend to prefer surgery when studying abroad in high-income countries (39), reflecting the influence of external environments on career choice preferences.

Secondly, sex-based differences were observed, with male students demonstrating a higher preference for surgery, while female students were more inclined toward medicine. These findings are consistent with local and global trends, as gender-related factors such as lifestyle priorities, societal expectations, and perceived barriers which play a significant role in shaping specialty preferences are similar around the globe (23, 28, 40–42). Males are often attracted to the prestige, technical appeal, and earning potential of surgery, while females may prefer medicine for its flexibility, continuity of care, and better work–life balance (42–44). Sociocultural expectations and underrepresentation of women in surgery further reinforce these patterns. These findings emphasize the influence of gender-related factors on specialty choices and the importance of fostering equitable opportunities across disciplines.

Institutional type also influenced career preferences, with students from private institutions exhibiting a stronger preference for surgery compared to their counterparts in government institutions, which is line with the findings from Mahsood et al., study (19). This atypical finding may be attributed to sectoral differences in a range of factors identified by locally conducted studies (44, 45), which encompass institutional and societal culture, mentorship opportunities, personal experiences, family obligations, and access to resources, as well as variations in mentorship opportunities, exposure to sub-specialties, competition for postgraduate training, and socioeconomic background (23).

Moreover, our study also highlighted the dominant factors influencing specialty preference such as personal interest, perceived career opportunities, and clinical rotations (5–8, 13, 46), underscoring the importance of intrinsic motivation and experiential learning in specialty choice. Additionally, discouragement factors such as lack of mentorship, gender inequity, and family obligations also play a significant role, highlighting the complex interplay of personal, professional, and societal influences on career specialty choices among medical students (20, 46, 47). Taken together, these findings mirror patterns across the globe from Low to high income economies (13, 22–30, 32, 37). Personal interest, exposure through clinical rotations, and anticipated career prospects often drive specialty choices, while barriers such as inadequate mentorship, gender inequity, and family obligations act as strong deterrents (46, 48, 49). This reflects the balance between intrinsic motivation and external constraints that shape students’ decisions.

Moreover, for both surgery and medicine, the primary factors influencing career choice were personal interest and clinical rotations. Personal interest reflects an individual’s passion for the field, while clinical rotations allow students to gain firsthand exposure to different specialties, helping them make informed decisions (50). However, for surgery, the availability of diverse career opportunities emerged as the second most significant factor (51). This reflects the broader spectrum of subspecialties within surgery, which offers surgeons the flexibility to specialize in various areas, such as orthopedics, neurosurgery, or cardiovascular surgery. On the other hand, lifestyle considerations played a more prominent role in the choice of medicine. This is likely due to the traditionally more predictable work hours and the potential for a more balanced work-life dynamic in medicine, compared to the often demanding and unpredictable nature of surgical practice (52). These factors highlight the varying personal and professional priorities that guide career decisions within these two fields, which are consistent across sex, educational years and institutional sector.

Furthermore, the three most significant discouragement factors for both surgery and medicine included a lack of mentorship, gender inequity, and family obligations, as consistently reported across sex, education year, and institutional sector. A lack of mentorship emerged as a universal barrier, reflecting the critical need for structured guidance during clinical years to facilitate career exploration and skill-building (47, 52). Gender inequity was particularly notable in surgery, where the underrepresentation of women and limited access to supportive networks perpetuate disparities, potentially discouraging female students (20). Family obligations, a shared challenge for both specialties, often weigh more heavily on female students due to societal expectations around caregiving roles, thereby influencing career choices (47, 53). The trends observed across education years suggest that these deterrents remain significant throughout the clinical years, indicating the persistent nature of these challenges. Institutional sector also played a role, with students from government institutions reporting unique influences; for instance, colleagues and friends emerged as the second most significant deterrent for pursuing surgery, highlighting the impact of peer competition and societal perceptions, which has not been highlighted in prior literature. This finding is particularly significant in Pakistan, where cultural norms and a highly competitive academic environment in government medical institutions strongly shape career decisions (54). These findings collectively underscore the multifaceted nature of career deterrents in surgery and medicine, influenced by personal, societal, and institutional factors, and emphasize the need for tailored interventions to improve mentorship, reduce gender inequities, and support students in managing family responsibilities.

This study has several limitations that merit consideration. One of the limitations of this study is its cross-sectional design, which captures students’ preferences at a single point in time, limiting the ability to infer causality or track how factors influencing specialty choices evolve throughout medical training, and long-term studies are recommended and needed. Furthermore, the study was conducted in Pakistan, which may limit the generalizability of its findings to medical students internationally, where cultural and institutional differences could shape specialty choices. Cultural norms and systemic structures vary widely across South Asia and globally. For example, differences in the prestige of specialties, family expectations, and the balance between private and public medical education systems could lead to distinct trends in other contexts. Thus, while the results provide valuable insights into the Pakistani medical education landscape, they should be interpreted with caution when applied to other regions. Comparative studies across multiple provinces and countries with similar socio-economic and educational frameworks (e.g., India, Nepal, Bangladesh) are warranted to validate whether these trends are consistent or context-specific. The use of self-reported data and face to face data collection may introduces potential biases, including recall bias and social desirability bias, which may impact the accuracy of responses regarding career specialty selection and influencing factors and deterrents. Although efforts were made to enhance the validity of the questionnaire, certain influential factors may have been overlooked, such as the quality of institutional mentorship or family dynamics, might not have been adequately captured. Additionally, the sampling strategy, while random, may not fully account for variations in unmeasured variables, such as socioeconomic status or preclinical exposures. Finally, the study did not examine longitudinal changes in specialty preferences post-graduation, which could provide deeper insights into the evolution of career decision-making processes over time. Future research should not only employ longitudinal designs to track how preferences evolve across medical training and into postgraduate practice, but also examine whether the observed associations (e.g., mentorship gaps, gender inequity) persist over time and influence actual career outcomes.

In conclusion, this study highlights a significant trend in the career specialty preferences of clinical-year medical students in Karachi, Pakistan. The data reveal a notable shift from surgery to medicine as students progressed through their education, indicating an evolving outlook on career choices. The trend was influenced by a variety of factors, with personal interest, perceived career opportunities, and exposure through clinical rotations being the most prominent drivers of specialty selection. On the other hand, several discouragement factors such as a lack of mentorship, gender inequity, and family obligations emerged as substantial barriers to pursuing specific fields. These findings underscore the importance of addressing these factors by promoting mentorship programs, reducing gender-based biases, and providing better support for students facing familial constraints. By addressing these challenges, medical institutions can foster more informed and diverse specialty choices, ultimately contributing to a more balanced distribution of healthcare professionals across various specialties in Pakistan. Moreover, this study serves as a valuable foundation for exploring similar trends in other countries, offering insights that can help inform medical education policies and specialty distribution strategies globally.

## Data Availability

The datasets presented in this article are not readily available in order to safeguard participant anonymity and uphold the confidentiality of responses, data are available from the corresponding author on reasonable request, with identifiable information removed. Requests to access the datasets should be directed to MD, muhammadhamzadawood86@gmail.com.

## References

[1] Mirvis DM. Choosing a medical specialty: the difference between what students want and what society needs. Isr J Health Policy Res. (2013) 2:18. doi: 10.1186/2045-4015-2-18, PMID: 23692643 PMC3665462

[2] Yang Y Li J Wu X Wang J Li W Zhu Y. Factors influencing subspecialty choice among medical students: a systematic review and meta-analysis. BMJ Open. (2019) 9:e022097. doi: 10.1136/bmjopen-2018-022097, PMID: 30850399 PMC6429728

[3] Elgasim M Omran OM. Factors affecting the choice of specialty among undergraduate medical students, Qassim university, 2020. Int J Med Res & Health Sci. (2021) 10:132–40.

[4] Guraya SY Almaramhy HH. Mapping the factors that influence the career specialty preferences by the undergraduate medical students. Saudi J Biol Sci. (2018) 25:1096–101. doi: 10.1016/j.sjbs.2017.03.019, PMID: 30174508 PMC6117166

[5] Kuteesa J Musiime V Munabi IG Mubuuke AG Opoka R Mukunya D. Specialty career preferences among final year medical students at Makerere University College of health sciences, Uganda: a mixed methods study. BMC Med Educ. (2021) 21:215. doi: 10.1186/s12909-021-02630-x, PMID: 33863332 PMC8052684

[6] Mohamed EY. Specialty preferences and factors affecting the choices of postgraduate specialty among undergraduate medical students. Pak J Med Sci. (2022) 38:1431–5. doi: 10.12669/pjms.38.6.5571, PMID: 35991256 PMC9378383

[7] Sawan D Alrefaei GM Alesawi A Abualross O Alsuwaida SA Meer N. Preferences, career aspects, and factors influencing the choice of specialty by medical students and interns in Saudi Arabia: a cross-sectional study. Cureus. (2023) 15:e43018. doi: 10.7759/cureus.43018, PMID: 37674943 PMC10478148

[8] Abdulrahman M Makki M Shaaban S Al Shamsi M Venkatramana M Sulaiman N. Specialty preferences and motivating factors: a national survey on medical students from five Uae medical schools. Educ Health (Abingdon). (2016) 29:231–43. doi: 10.4103/1357-6283.204225, PMID: 28406108

[9] Grasreiner D Dahmen U Settmacher U. Specialty preferences and influencing factors: a repeated cross-sectional survey of first- to sixth-year medical students in Jena, Germany. BMC Med Educ. (2018) 18:103. doi: 10.1186/s12909-018-1200-8, PMID: 29743057 PMC5944057

[10] Alsubaie N Aldhofaian HS Alhuwaimel L Ruxshan N Alghamdi F Shamia A. Specialty preferences and the factors influencing them among pre-clerkship medical students: the first study from Alfaisal University-College of Medicine, Saudi Arabia. Cureus. (2016) 8:e894. doi: 10.7759/cureus.894, PMID: 28018764 PMC5179192

[11] Alawad AA Khan WS Abdelrazig YM Elzain YI Khalil HO Ahmed OB. Factors considered by undergraduate medical students when selecting specialty of their future careers. Pan Afr Med J. (2015) 20:102. doi: 10.11604/pamj.2015.20.102.4715, PMID: 26090050 PMC4458322

[12] Alyazidi AS Gaddoury MA Alotibi FA Aljehani KM Ahmed RA Alhudaifi SA. The determining factors of medical students in considering a specialty as a future career path: a cross-sectional multinational study in the Middle East. J Family Med Prim Care. (2023) 12:2622–34. doi: 10.4103/jfmpc.jfmpc_1742_22, PMID: 38186787 PMC10771143

[13] Anand R Sankaran PS. Factors influencing the career preferences of medical students and interns: a cross-sectional, questionnaire-based survey from India. J Educ Eval Health Prof. (2019) 16:12. doi: 10.3352/jeehp.2019.16.12, PMID: 31117329 PMC6609296

[14] Leutritz T Krauthausen M Simmenroth A König S. Factors associated with medical students' career choice in different specialties: a multiple cross-sectional questionnaire study at a German medical school. BMC Med Educ. (2024) 24:798. doi: 10.1186/s12909-024-05751-1, PMID: 39049024 PMC11270969

[15] Asiri WMA Shati AA Alrowaibah NA Althumairi RK Alqahtani GM Mahmood SE. The influencing factors of choosing future medical specialties among students in Saudi Arabia: a nationwide multicenter survey. Medicine (Baltimore). (2023) 102:e33483. doi: 10.1097/MD.0000000000033483, PMID: 37026904 PMC10082225

[16] Dawod MS Alswerki MN Al-Takhaineh MA. Factors that influence medical students' decision to pursue a career in orthopaedics: a comprehensive analysis. Int Orthop. (2024) 48:1139–47. doi: 10.1007/s00264-024-06132-5, PMID: 38436709

[17] Rehman A Rehman T Shaikh MA Yasmin H Asif A Kafil H. Pakistani medical students' specialty preference and the influencing factors. J Pak Med Assoc. (2011) 61:713–8. 22204259

[18] Ehsan SB. Factors influencing medical students' choice for family medicine as a specialty in Pakistan. J Ayub Med Coll Abbottabad. (2018) 30:203–8. 29938419

[19] Mahsood YJ Rehman A Aman T Abid S Hashim N Ayub R. Comparison of changes in Speciality choices and future career plans between public and private medical students over five years of medical studies. J Coll Physicians Surg Pak. (2023) 33:705–8. doi: 10.29271/jcpsp.2023.06.705, PMID: 37300270

[20] Dawood MH Roshan M Daniyal M Sohail S Perveen H Islam UU. Gender inequity in clinical clerkships and its influence on career selection: a cross-sectional survey. J Med Educat Curri Develop. (2024) 11:23821205241257401. doi: 10.1177/23821205241257401, PMID: 38799175 PMC11128173

[21] Georgiev G.Z. "Random Name Picker" [Internet]. Available online at: https://www.gigacalculator.com/randomizers/random-name-picker.php (Accessed August 6, 2024).

[22] Iktidar MA Sakib MM Munni UR Rimti FH Yousuf R Majumder K. Medical students’ career preferences in Bangladesh. BMC Med Educ. (2024) 24:81. doi: 10.1186/s12909-024-05050-9, PMID: 38263095 PMC10804597

[23] Khan Q Rao M Anwar H Qamar M Hafeezz F Asif S. Preference of specialty in medical students of private and government medical colleges. PJMHS (Pakistan Journal of Medical & Health Sciences). (2022). Lahore, Pakistan.

[24] Querido S De Rond M Wigersma L van den Broek S ten Cate O. The significance of experiencing clinical responsibilities for specialty career choice. Med Sci Educ. (2020) 30:163–71. doi: 10.1007/s40670-019-00832-z, PMID: 34457655 PMC8368942

[25] Coffeng LE Visscher AJE Cate OTJT. The influence of early clinical experiences on career preference of male and female medical students. Med Teach. (2009) 31:e323–6. doi: 10.1080/01421590802650084, PMID: 19811141

[26] Shakir M Irshad HA Ali EA Adil A Altaf A Enam SA. Impact of medical school experiences on the career choice of neurosurgery: a cross- sectional study from Pakistan. BMC Med Educ. (2024) 24:465. doi: 10.1186/s12909-024-05452-9, PMID: 38671453 PMC11055371

[27] Rachoin J-S Vilceanu MO Franzblau N Gordon S Cerceo E. How often do medical students change career preferences over the course of medical school? BMC Med Educ. (2023) 23:596. doi: 10.1186/s12909-023-04598-2, PMID: 37608363 PMC10463921

[28] Jha RK Paudel KR Shah DK Sah AK Basnet S Sah P. Subject preferences of first- and second-year medical students for their future specialization at Chitwan medical college and teaching hospital, Chitwan, Nepal - a questionnaire-based study. Adv Med Educ Pract. (2015) 6:609–13. doi: 10.2147/AMEP.S92534, PMID: 26635491 PMC4646476

[29] Ossai EN Uwakwe KA Anyanwagu UC Ibiok NC Azuogu BN Ekeke N. Specialty preferences among final year medical students in medical schools of Southeast Nigeria: need for career guidance. BMC Med Educ. (2016) 16:259. doi: 10.1186/s12909-016-0781-3, PMID: 27716155 PMC5050581

[30] Khader Y Al-Zoubi D Amarin Z Alkafagei A Khasawneh M Burgan S. Factors affecting medical students in formulating their specialty preferences in Jordan. BMC Med Educ. (2008) 8:32. doi: 10.1186/1472-6920-8-32, PMID: 18501004 PMC2423351

[31] Godbole AA Oka GA Ketkar MN Solanki RS Desai DT Bangale SV. Specialty preferences of undergraduate medical students: what do they choose and why? Med J Armed Forces India. (2025) 81:66–71. doi: 10.1016/j.mjafi.2024.04.009, PMID: 39872182 PMC11762663

[32] Creed PA Searle J Rogers ME. Medical specialty prestige and lifestyle preferences for medical students. Soc Sci Med. (2010) 71:1084–8. doi: 10.1016/j.socscimed.2010.06.027, PMID: 20674118

[33] Gawad N Moussa F Christakis GT Rutka JT. Planting the 'SEAD': early comprehensive exposure to surgery for medical students. J Surg Educ. (2013) 70:487–94. doi: 10.1016/j.jsurg.2013.03.006, PMID: 23725936

[34] Al-Faifi JJ Alsarar SA Bayamin RA Alkhaldi RA Hawsawi HS Alromih AM. Factors influencing medical students' decision in choosing a surgical specialty. Cureus. (2024) 16:e70416. doi: 10.7759/cureus.70416, PMID: 39473681 PMC11521400

[35] Peel JK Schlachta CM Alkhamesi NA. A systematic review of the factors affecting choice of surgery as a career. Can J Surg. (2018) 61:58–67. doi: 10.1503/cjs.008217, PMID: 29368678 PMC5785290

[36] Nguyen QT Luong TTH Nguyen HV Ngo MQ Nguyen SH Nguyen NLK. Aspirations to study medicine, perceptions of a good doctor, and their influence on specialty choice among medical students. PLoS One. (2025) 20:e0326266. doi: 10.1371/journal.pone.0326266, PMID: 40526726 PMC12173351

[37] Lefevre JH Roupret M Kerneis S Karila L. Career choices of medical students: a national survey of 1780 students. Med Educ. (2010) 44:603–12. doi: 10.1111/j.1365-2923.2010.03707.x, PMID: 20604857

[38] Puertas EB Arósquipa C Gutiérrez D. Factors that influence a career choice in primary care among medical students from high-, middle-, and low-income countries: a systematic review. Rev Panam Salud Publica. (2013) 34:351–8. PMID: 24553763

[39] Li W Gillies RM Liu C Wu C Chen J Zhang X. Specialty preferences of studying-abroad medical students from low- and middle-income countries. BMC Med Educ. (2023) 23:158. doi: 10.1186/s12909-023-04123-5, PMID: 36922811 PMC10015544

[40] Asaad M Zayegh O Badawi J Hmidi Z Alhamid A Tarzi M. Gender differences in specialty preference among medical students at Aleppo university: a cross-sectional study. BMC Med Educ. (2020) 20:184. doi: 10.1186/s12909-020-02081-w, PMID: 32503519 PMC7275529

[41] Yin K Yang L Zhang R Zheng D Wilkes MS Lai Y. Gender differences and influencing factors in specialty choices: findings from one medical School in China. Front Public Health. (2021) 9:648612. doi: 10.3389/fpubh.2021.648612, PMID: 33842425 PMC8027487

[42] Lee CW. Gender difference and specialty preference in medical career choice. Korean J Med Educ. (2013) 25:15–21. doi: 10.3946/kjme.2013.25.1.15, PMID: 25804649 PMC8813412

[43] Diderichsen S Johansson EE Verdonk P Lagro-Janssen T Hamberg K. Few gender differences in specialty preferences and motivational factors: a cross-sectional Swedish study on last-year medical students. BMC Med Educ. (2013) 13:39. doi: 10.1186/1472-6920-13-39, PMID: 23497262 PMC3599519

[44] Burgos CM Josephson A. Gender differences in the learning and teaching of surgery: a literature review. Int J Med Educ. (2014) 5:110–24. doi: 10.5116/ijme.5380.ca6b, PMID: 25341220 PMC4207172

[45] Zaheer F Rehman HU Fareed W Khan MO Rizvi SAH. Factors affecting the choice of a career in the field of surgery among medical students of Karachi. Cureus. (2018) 10:e3542. doi: 10.7759/cureus.3542, PMID: 30648075 PMC6324860

[46] Sarikhani Y Ghahramani S Bayati M Lotfi F Bastani P. A thematic network for factors affecting the choice of specialty education by medical students: a scoping study in low-and middle-income countries. BMC Med Educ. (2021) 21:99. doi: 10.1186/s12909-021-02539-5, PMID: 33568113 PMC7877062

[47] Trinh LN O'Rorke E Mulcahey MK. Factors influencing female medical students' decision to pursue surgical specialties: a systematic review. J Surg Educ. (2021) 78:836–49. doi: 10.1016/j.jsurg.2020.08.050, PMID: 32933885

[48] Nguyen QT Bui NY Nguyen MPN Nguyen HV Thuy MH. Do structured career counselling initiatives influence specialty preferences in medical students? A longitudinal observational survey study. BMJ Open. (2025) 15:e099815. doi: 10.1136/bmjopen-2025-099815, PMID: 40374226 PMC12083375

[49] Nguyen QT Nguyen MPN Bui NY Ngo MQ Nguyen LVT Dang TT. The complex interplay of personal and external factors in medical students' specialty decision-making: a qualitative study. PLoS One. (2025) 20:e0326932. doi: 10.1371/journal.pone.0326932, PMID: 40569991 PMC12200644

[50] Bechara JP Shah PP Lindor K. The power of rotation schedules on the career selection decisions of medical students. Adv Health Sci Educ Theory Pract. (2023) 28:1509–22. doi: 10.1007/s10459-023-10227-w, PMID: 37131109 PMC10153029

[51] Glynn RW Kerin MJ. Factors influencing medical students and junior doctors in choosing a career in surgery. Surgeon. (2010) 8:187–91. doi: 10.1016/j.surge.2009.11.005, PMID: 20569936

[52] Alshahrani M Dhafery B Al Mulhim M Alkhadra F Al Bagshi D Bukhamsin N. Factors influencing Saudi medical students and interns' choice of future specialty: a self-administered questionnaire. Adv Med Educ Pract. (2014) 5:397–402. doi: 10.2147/AMEP.S69152, PMID: 25368542 PMC4216024

[53] Ruparell K Barve R Tas RN Chen S McLaughlin R Ravendren A. Motivators and deterrents for early career female doctors applying to surgical training programmes in the UK National Health Service: a mixed-methods study. BMJ Open. (2022) 12:e055652. doi: 10.1136/bmjopen-2021-055652, PMID: 36456020 PMC9723904

[54] Lee MH Iyengar Y Budiansky D Veinot P Law M. Exploring medical students' perceptions of peer-to-peer interactions related to applying to a surgical residency. J Surg Educ. (2024) 81:193–201. doi: 10.1016/j.jsurg.2023.11.007, PMID: 38142152

