# Supplementary material for: A cross-sectional investigation of trend in career specialty preference among clinical year medical undergraduates, including factors influencing preferences and discouragement
Source: Front Med (Lausanne). 2025 Oct 17;12:1665043. doi: 10.3389/fmed.2025.1665043 (PMC12576040; doi:10.3389/fmed.2025.1665043)
Supplement: Supplementary file 1 [file Data_Sheet_1.docx]

**INFORMED CONSENT FORM**

You are being invited to participate in a cross-sectional study designed having the title, **"** **A Cross-sectional Investigation of Trend in Career Specialty Preference Among Clinical Year Medical Undergraduates, including Factors Influencing Preferences and Discouragement."**

This study is being conducted by Students at United Medical and Dental College (UMDC) with the help of its Surgery Department.

This questionnaire contains short relevant close & open-ended questions, which will take around 5 minutes to fill. Your participation in this research will be valuable for us.

The information you provide will remain confidential and will only be used in summary statistics.

There is no risk(s) or discomfort is involved.

***DISCLAIMER:***

There is no financial compensation for your participation in this study.

**Kindly read and consent to the following statement:**

1. My participation in this study is completely voluntary and I can withdraw at any time.

2. I understand that answers recorded will be completely anonymous.

3. I understand that the information from my responses will be used for academic research.

4. I give my full consent to participate in this study.

5. The information provided by myself will be of my honest will.

**Do you wish to participate? * Yes No**

**Name*: ________________________________________ Signature of Participant: _____________________**

**QUESTIONNAIRE**

***Part-I: Student's Demographic Information:***

1. **Email address (Confidential/Optional): _______________________________________________________**
2. **Age * ____________**
3. **Tick the appropriate box:**
4. **Sex *** Male Female
5. **Current Education status ***
6. 3^rd^ Year 4^th^ Year 5^th^ year
7. **University/College Name ***

| HIDDEN DUE TO CONFIDENTIALITY | 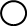 |
| --- | --- |
| HIDDEN DUE TO CONFIDENTIALITY | 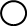 |
| HIDDEN DUE TO CONFIDENTIALITY | 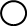 |
| HIDDEN DUE TO CONFIDENTIALITY | 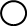 |
| HIDDEN DUE TO CONFIDENTIALITY | 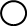 |
| HIDDEN DUE TO CONFIDENTIALITY | 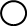 |
| HIDDEN DUE TO CONFIDENTIALITY | 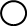 |
| HIDDEN DUE TO CONFIDENTIALITY | 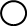 |
| HIDDEN DUE TO CONFIDENTIALITY | 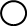 |
| HIDDEN DUE TO CONFIDENTIALITY | 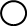 |
| HIDDEN DUE TO CONFIDENTIALITY | 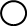 |
| HIDDEN DUE TO CONFIDENTIALITY | 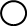 |
| HIDDEN DUE TO CONFIDENTIALITY | 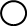 |
| HIDDEN DUE TO CONFIDENTIALITY | 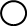 |
| HIDDEN DUE TO CONFIDENTIALITY | 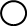 |

***Part-II: Questions about your career preference***

(Surgery and medicine are two broad terms that cover all different types of medical sub-specialties from which we have to opt while starting our residency program. This decision is influenced by many factors. Based on your experience, please answer the following questions to your honest will.)

**A1- Which Specialty do you intend to pursue as your future career? ***

Medicine (PHYSICIAN). Surgery (SURGEON).

**A2- Why are you opting this specific specialty as your future career?** ***** *(Mark all which fits for you based on your answer to A1)*

| Own interest/Passion. |  |
| --- | --- |
| Mentorship. |  |
| Peer pressure/ Social pressure/ Friendship preferences. |  |
| Clinical Rotation. |  |
| Vast career opportunities. |  |
| Lifestyle. |  |
| Trending. |  |
| Family Decision. |  |
| Other: __________________________________ (Specify) |  |

**A3- What factors are influencing your decision to not pursue other medical specialty?** * (Mark All which applies to you, *based on your answer to A1*).

| Lack of mentorship |  |
| --- | --- |
| Societal/Institutional/Cultural Norms |  |
| Family obligations |  |
| Less career opportunities |  |
| Lifestyle |  |
| Gender inequity |  |
| Colleagues/Friends |  |
| Social media Trends |  |
| Traditional stereotypes |  |
| None |  |
| Other: ____________________________________ (Specify) |  |
